# Pab1 acetylation at K131 decreases stress granule formation in *Saccharomyces cerevisiae*

**DOI:** 10.1016/j.jbc.2022.102834

**Published:** 2022-12-24

**Authors:** Sangavi Sivananthan, Jessica T. Gosse, Sylvain Huard, Kristin Baetz

**Affiliations:** 1 Ottawa Institute of Systems Biology, Department of Biochemistry, Microbiology and Immunology, University of Ottawa, Ottawa, Ontario, Canada; 2 Department of Biological Sciences, University of Calgary, Calgary, Alberta, Canada

**Keywords:** acetylation, *Saccharomyces cerevisiae*, stress granule, KAT, KDAC, KAT, lysine acetyltransferase, KDAC, lysine deacetylase, Pab1, poly(A)-binding protein 1, RRM, RNA recognition motif, SC, Synthetic Completion media without glucose, SCD, Synthetic Complete Dextrose media with glucose, SG, stress granule, YPD, yeast peptone dextrose

## Abstract

Under environmental stress, such as glucose deprivation, cells form stress granules—the accumulation of cytoplasmic aggregates of repressed translational initiation complexes, proteins, and stalled mRNAs. Recent research implicates stress granules in various diseases, such as neurodegenerative diseases, but the exact regulators responsible for the assembly and disassembly of stress granules are unknown. An important aspect of stress granule formation is the presence of posttranslational modifications on core proteins. One of those modifications is lysine acetylation, which is regulated by either a lysine acetyltransferase or a lysine deacetylase enzyme. This work deciphers the impact of lysine acetylation on an essential protein found in *Saccharomyces cerevisiae* stress granules, poly(A)-binding protein (Pab1). We demonstrated that an acetylation mimic of the lysine residue in position 131 reduces stress granule formation upon glucose deprivation and other stressors such as ethanol, raffinose, and vanillin. We present genetic evidence that the enzyme Rpd3 is the primary candidate for the deacetylation of Pab1-K131. Further, our electromobility shift assay studies suggest that the acetylation of Pab1-K131 negatively impacts poly(A) RNA binding. Due to the conserved nature of stress granules, therapeutics targeting the activity of lysine acetyltransferases and lysine deacetylase enzymes may be a promising route to modulate stress granule dynamics in the disease state.

When exposed to environmental stress, eukaryotic cells utilize several cellular mechanisms to remodel their pathways to specifically respond to stress and increase survival. One of these response mechanisms is the formation of cytoplasmic RNA-protein aggregates, or stress granules (SGs), which develop under a variety of acute stress conditions such as glucose deprivation, presence of toxins, and diseases. SGs are composed of stalled mRNA, poly(A)-binding proteins (Pab1), and other signaling molecules (1). The compartmentalization of these vital components promotes their protection and cell survival, and once the stressor is removed, protein translation can be resumed. Not only is the formation of SG required for cellular survival against various environmental challenges, but in humans, they are a hallmark for disease progression of many neurodegenerative diseases and cancers (2, 3, 4, 5).

Several signaling pathways have been implicated in regulating SG assembly and disassembly (6); however, the molecular details of these pathways remain largely unknown. A signaling axis that contributes to SG dynamics is the posttranslational modification of amino acid residues, including lysine acetylation (7). In an enzyme-mediated process, lysine acetyltransferases (KATs) add an acetyl moiety to lysine residues (8, 9), while lysine deacetylases (KDACs) catalyze the removal of the acetyl moiety (10). Acetylation modifies the charge and structure of the lysine residue, thus altering the interactions with other cellular components.

KATs and KDACs have been implicated in both the regulation and inhibition of SG formation in yeast. Mutants of the NuA4 KAT complex had decreased SG formation upon glucose deprivation but showed no change upon exposure to other stressors (11). Systematic screening of deletion mutants of nonessential KAT and KDAC enzymes demonstrated that KAT mutants Gcn5, Rpd3, and Hos3 and KDAC mutant Hst1 displayed increased SG formation under nonstress conditions in yeast (11, 12). Further, the analysis of numerous yeast and human homologs, KATs and KDACs, shows that they colocalize in SGs, suggesting a target within SGs (13, 14). In agreement with this, large-scale acetylome studies detected multiple lysine acetylation sites on many proteins found in SGs, including Pab1. Acetylation has been detected on several Pab1 lysine (K) sites, including K7, K131, K164, K288, and K504 and which suggests that Pab1 might be a target for KATs and KDACs once inside the SG (15, 16, 17, 18).

RNA recognition motif (RRM) is a common protein domain with roles in posttranscriptional processes such as pre-mRNA processing, mRNA nuclear export, translational regulation, and mRNA decay (19, 20, 21). Pab1 has four RRMs that are highly conserved among the cytoplasmic Pab1 family of proteins. The RRM domains associate directly with RNA and mediate protein–protein interactions. For example, Pab1 RRM2 domain mediate the association with eIF4G initiation complex that is required for the closed loop structure between the mRNA cap and poly(A) tail for translation (22, 23, 24, 25, 26). Pab1 RRM1 and RRM2 are both important for the binding of the poly(A) tail, with RRM2 has the highest affinity and specificity for poly(A) RNA (27). Under stress conditions, Pab1 presents a protecting role where mRNA poly(A) tails bind to the RRM motifs and remain in stable circular conformation, inhibiting deadenylase activity and preventing degradation due to recruitment of the complex into SGs (28, 29, 30, 31). This protective effect is observed during glucose deprivation (32), with strains lacking Pab1 presenting reduction in SG formation (1).

Herein, we systematically mutated known Pab1 lysine acetylation site and identified that K131 presents a role in SG dynamics. Our work suggests that the acetylation of Pab1-K131 inhibits interaction with the poly(A) RNA tail and the formation of glucose-deprived SGs, showing that this site must be deacetylated for efficient formation of SGs and further protection of the translation machinery. As Pab1-K131 is a conserved site found in many Pab1, lysine acetylation at this position could be a mechanism to control not just SG dynamics but poly(A) RNA binding. Given that SGs have been implicated in a wide variety of diseases, in determining the molecular mechanisms regulating the formation and disassembly of SGs using yeast as a model, we can reveal novel potential therapeutic targets.

## Results

### Pab1-K131 mutation impacts glucose-deprived SG formation

**Figure 1:**
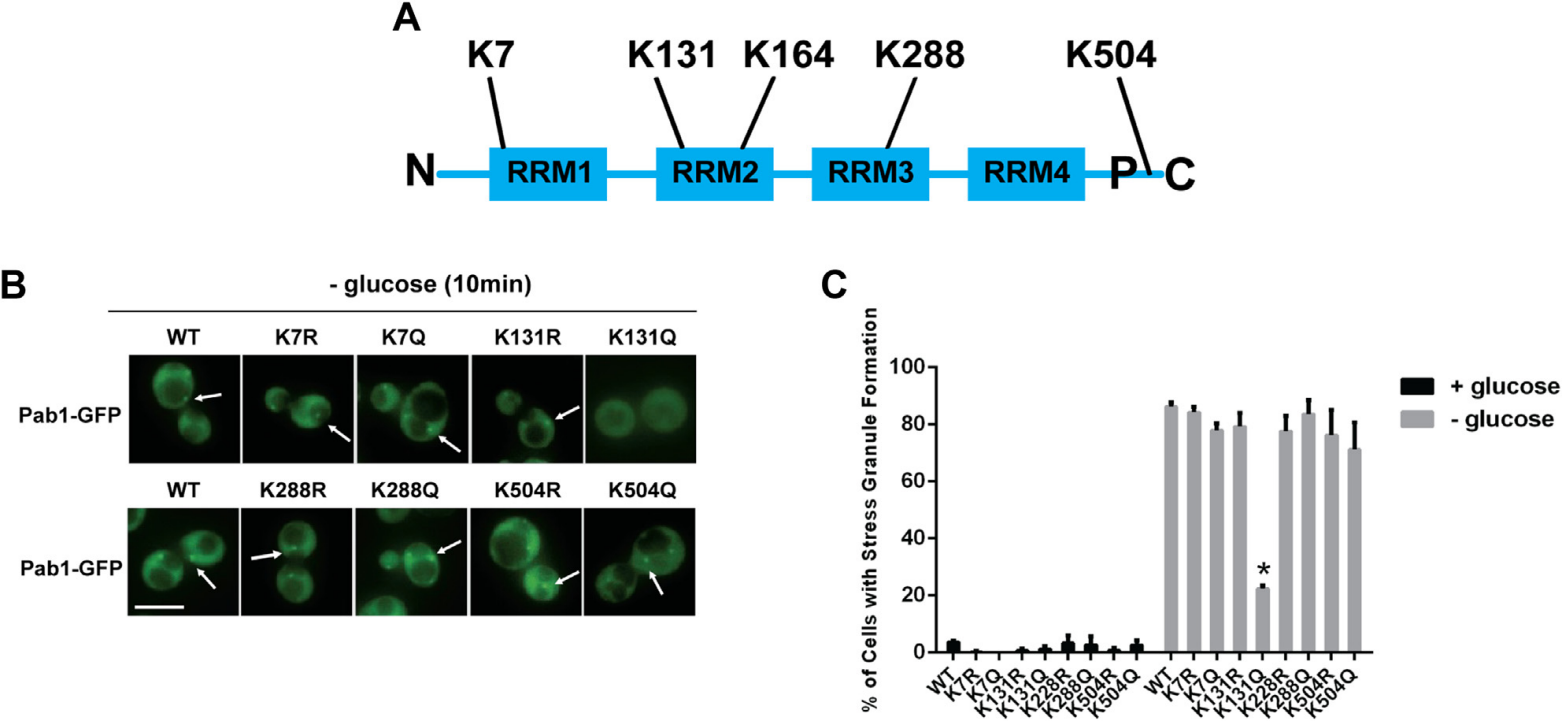
**Formation of stress granules is reduced in cells expressing *PAB1-K131Q-GFP*.***A*, a graphical representation of the Pab1 protein that includes the RNA recognition motifs (RRMs) and known acetylation sites. N, N-terminus; C, C-terminus; P, P-domain. *B*, *pab1Δ* cells were transformed with *PAB1-GFP::URA::CEN* plasmid (WT YKB4033) or plasmids expressing the indicated mutations *PAB1-K7R-GFP* (YKB4037), *PAB1-K7Q-GFP* (YKB4038), *PAB1-K131R-GFP* (YKB4035), *PAB1-K131Q-GFP* (YKB4036), *PAB1-K288R-GFP* (YKB4039), *PAB1-K288Q-GFP* (YKB4040), *PAB1-K504R-GFP* (YKB4041), and *PAB1-K504Q-GFP* (YKB4042). Strains were cultured in SCD-URA media at 30 °C and, exponential-phase cells were subjected to 10 min of glucose deprivation and immediately assessed for Pab1-GFP foci (stress granule). *Arrow* indicates SG. The scale bar represents 10 μm. *C*, quantification of the percentage of cells with stress granule formation after 10 min of glucose deprivation. Results are the average of three biological replicates, a minimum of 100 cells per replicate were scored, and the error bars indicate SEM. ∗*p* < 0.05 determined using a two-way ANOVA test. Pab1, poly(A)-binding protein 1; SCD, Synthetic Complete Dextrose media with gluco; SG, stress granule; URA, uracil.

**Figure 2:**
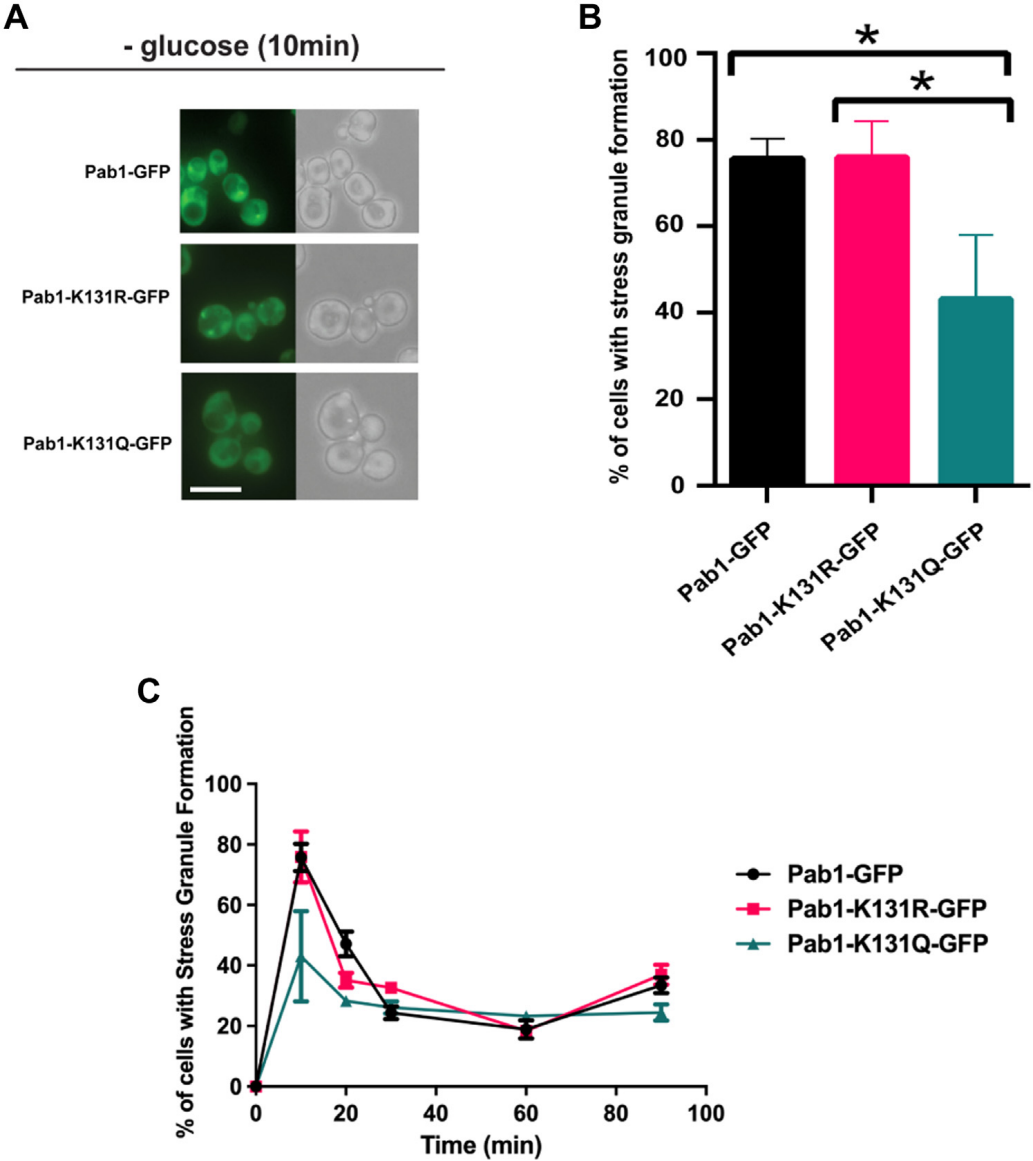
**Glucose-deprived stress granule formation is reduced in PAB1-K131Q-GFP mutant strain.** WT cells expressing endogenously tagged *PAB1-GFP* (YKB3114), *PAB1-K131R-GFP* (YKB4395), or *PAB1-K131Q-GFP* (YKB4396) were cultured in YPD media at 30 °C to mid-log phase (0 min), subjected to glucose deprivation for 10, 20, 30, 60, and 90 min, and immediately assessed for stress granules. *A*, representative brightfield and fluorescent images of cells after 10 min of glucose deprivation. The scale bar represents 10 μm. *B*, quantification of the percentage of cells with stress granule formation after 10 min of glucose deprivation. Results are the average of three biological replicates; a minimum of 100 cells per replicate were scored; the error bars indicate SEM. ∗*p* < 0.05 determined using a two-way ANOVA test. *C*, time course of the percentage of cells with stress granules at 10, 20, 30, 60, and 90 min. The results are the average of three biological replicates; a minimum of 100 cells per replicate were scored, and the error bars indicate SEM. Pab1, poly(A)-binding protein 1; YPD, yeast peptone dextrose; YP, yeast peptone.

To determine the impact of acetylation in Pab1, we tested four possible acetylation sites, K7, K131, K288, and K504, making either acetylated mimics (K to Q) or nonacetylated mimics (K to R) using a plasmid-based expression system where *PAB1* is tagged with GFP on its C-terminus (Fig. 1*A*). Multiple attempts failed to generate mutations at K164. We determined that cells expressing the acetylation mimic *PAB1-K131Q-GFP* displayed a reduction in glucose-deprived SGs (Fig. 1, *B* and *C*). To confirm these results were not an artifact of the plasmid system, CRISPR/Cas9 was used to create both mimics at *PAB1-GFP* expressed from its endogenous genomic location. While *PAB1-K131R-GFP* cells formed glucose-deprived SGs similarly to WT *PAB1-GFP* cells, *PAB1-K131Q-GFP* cells had a significant decrease in the formation of SGs after 10 min of glucose deprivation (Fig. 2, *A* and *B*). A time course experiment assessed SG formation after 10, 20, 30, 60, and 90 min of glucose deprivation. As expected, *PAB1-GFP* and *PAB1-K131R-GFP* cells formed glucose-deprived SGs and then slowly resolved the SGs over a 90-min time course. However, glucose-deprived SG formation remained low *in PAB1-K131Q-GFP* cells throughout the time course (Fig. 2*C*), indicating the reduction of SG formation in the K131 acetylation mimic is not reflective of a delay in SG formation. These results suggest that Pab1-GFP incorporation into glucose-deprived SGs is potentially regulated by the acetylation state of K131.

### Pab1-K131Q impacts SG formation upon ethanol, raffinose, and vanillin

**Figure 3:**
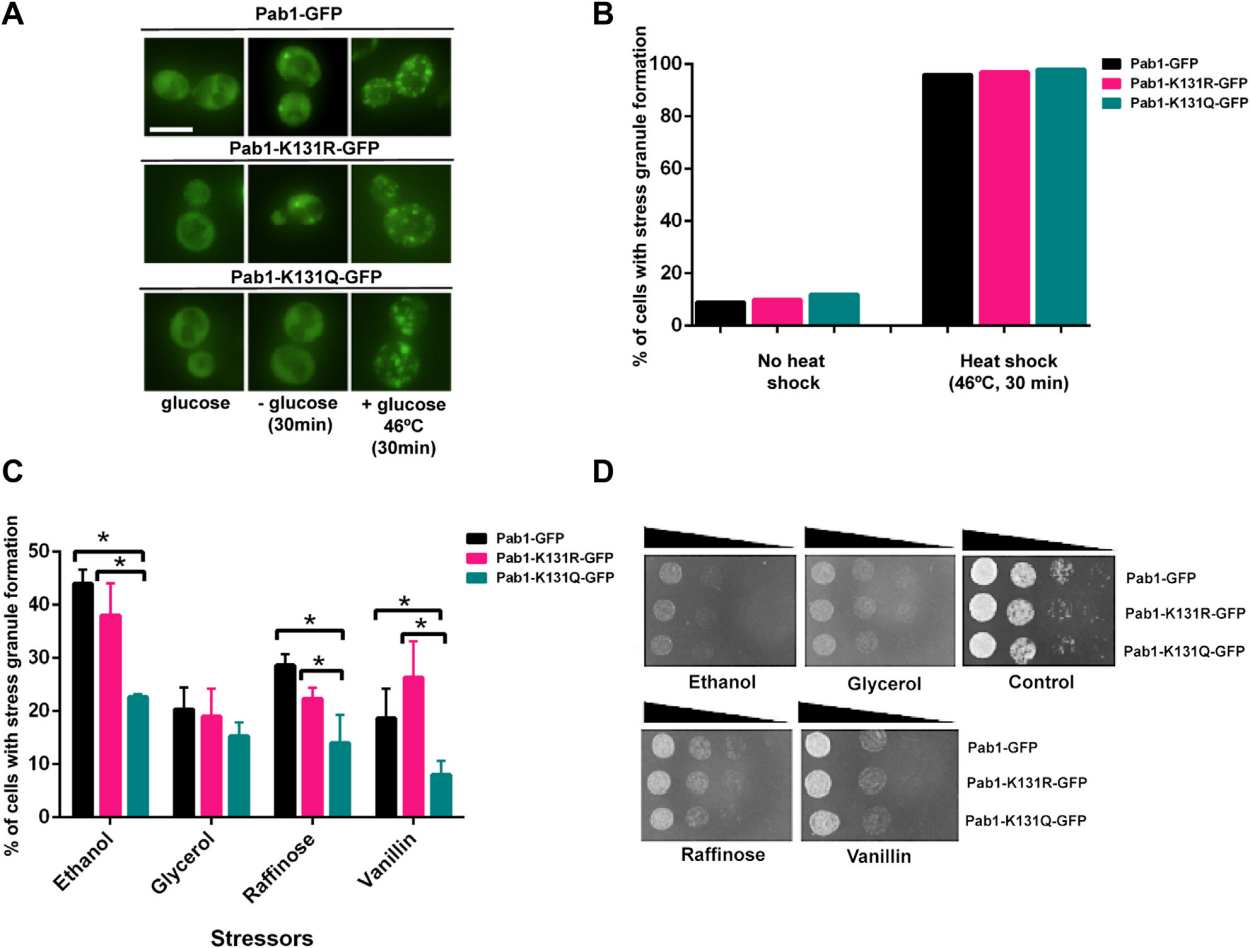
***S*tress granule formation upon ethanol, raffinose, and vanillin treatment is reduced in *PAB1-K131Q-GFP* cells.***A*, *PAB1-GFP* (YKB3114), *PAB1-K131R-GFP* (YKB4395), and *PAB1-K131Q-GFP* (YKB4396) expressing cells were cultured in YPD media at 30 °C and exponential-phase cells were subjected either to 30 min of glucose deprivation (YP) or heat shock at 46 °C in prewarmed YPD media. Representative fluorescent images are shown. The scale bar represents 10 μm. *B*, quantification of the percentage of cells with stress granule formation, with 30 min of heat shock at 46 °C. Results are average of three biological replicates; a minimum of 100 cells per replicate were scored, and the error bars indicate SEM. ∗*p* < 0.05 determined using a two-way ANOVA test. *C*, the indicated strains in *A* were cultured in YPD media at 30 °C and exponential-phase cells were subjected to 20 mM ethanol, 3% glycerol, 2% raffinose, and 30 nM vanillin for 30 min (see Experimental procedures for details). Quantification of the percentage of cells with stress granule formation after exposure to stress. Results average of three biological replicates, a minimum of 100 cells per replicate were scored, and the error bars indicate SEM. ∗*p* < 0.05 determined using a two-way ANOVA test. *D*, *PAB1-K131R-GFP* and *PAB1-K131Q-GFP* cells do not have detectable growth differences compared to *PAB1-GFP* cells. Dot assay of mutant mimics of K131 plated on YPD (*control*), YPD containing 30 nM vanillin, or YP containing 20 mM ethanol, 3% glycerol, and 2% raffinose. Plates were incubated at 30 °C for 24 h. The image is representative of three biological replicates. Pab1, poly(A)-binding protein 1; YPD, yeast peptone dextrose; YP, yeast peptone.

Previous research showed that a decrease in SG formation in NuA4 mutants was specific to glucose deprivation but that NuA4 did not impact SGs formed by other stressors such as ethanol or heat shock (11). Therefore, we sought to determine if Pab1-K131 acetylation mimics only impacted the formation of glucose-deprived SGs. Heat-induced SG formation was assessed after a 30-min heat shock at 46 °C and almost the totality of cells presented SGs regardless of acetylation mimic at K131 (Fig. 3, *A* and *B*). We extended the analysis to assess the impact of K131 mimics on chemically induced stress by incubating cells with ethanol, glycerol, raffinose, and vanillin. *PAB1-K131Q-GFP* cells showed a significant reduction in SG formation in 20 nM ethanol, 2% raffinose, and 30 nM vanillin but did not impact 3% glycerol-induced SG formation (Fig. 3*C*). Though *PAB1-K131Q-GFP* cells had a reduced rate of SG formation under these stressors, dot assays indicated this mutation does not result in detectable impacts on growth on agar plates containing ethanol, raffinose, or vanillin (Fig. 3*D*). This suggests that the acetylation state of Pab1-K131 impacts the SG formation only for a subset of cellular stresses, including glucose deprivation and chemically induced stress caused by ethanol, raffinose, and vanillin.

### Genetic screening identifies KDAC Rpd3 as potentially regulating Pab1 K131 acetylation sites

**Figure 4:**
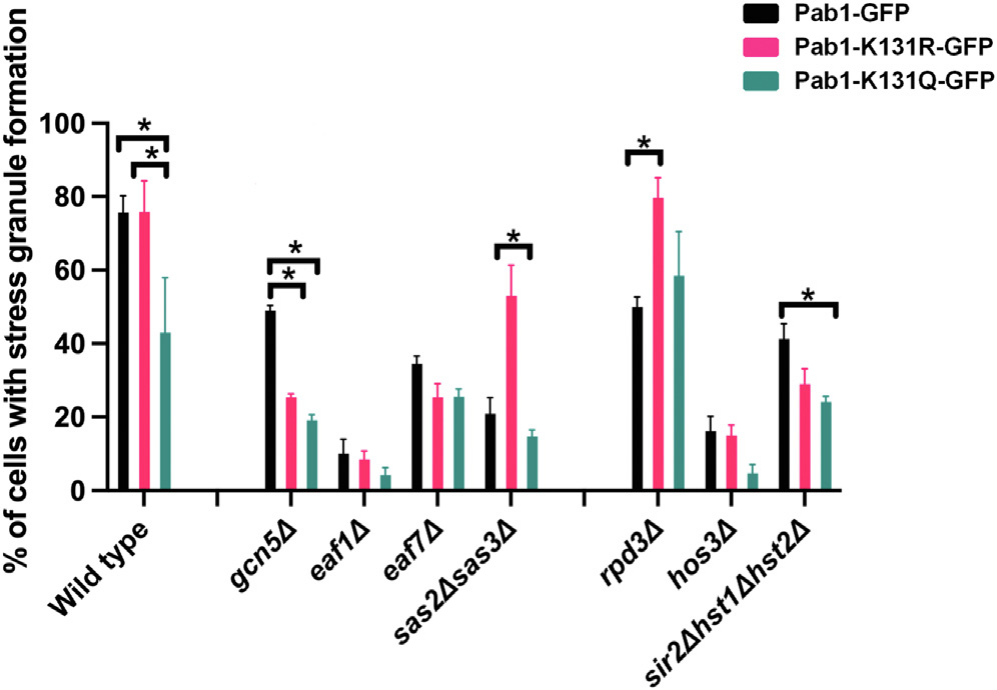
***PAB1-K131R-GFP* suppresses the reduction of glucose-deprived stress granule formation in *rpd3Δ* cells.** WT (YKB3114) and mutants *gcn5Δ* (YKB4177), *eaf1Δ* (YKB4175), *eaf7Δ* (YKB4969), *sas2Δsas3Δ* (YKB4161), *rpd3Δ* (YKB4180), *hos3Δ* (YKB4966), and *sir2Δhst1Δhst2Δ* (YKB4166) were transformed with plasmids expressing *PAB1-GFP* (PKB192), p*PAB1-K131R-GFP* (PKB355), and p*PAB1-K131Q-GFP* (PKB356). The strains were cultured in YPD media at 30 °C, and exponential-phase cells were subjected to 10 min of glucose deprivation in YP media and immediately assessed for SG formation. Results are the average of three biological replicates; a minimum of 100 cells per replicate were scored, and the error bars indicate SEM. ∗*p* < 0.05 determined using a two-way ANOVA test. Pab1, poly(A)-binding protein 1; SG, stress granule; YPD, yeast peptone dextrose; YP, yeast peptone.

We proposed a model where KAT(s) acetylating Pab1-K131 inhibits glucose-deprived SG formation while KDAC(s) deacetylating Pab1-K131 is required for SG formation. If this model were true, the mutant of the KAT(s) responsible for Pab1-K131 acetylation would present elevated glucose-deprived SG formation, due to decreased acetylation that would be suppressed by the *PAB1-K131Q-GFP* acetylation mimic. Similarly, KDAC mutant(s) responsible for the deacetylation of Pab1-K131 would have decreased glucose-deprived SG formation, due to increased Pab1-K131 acetylation and would be suppressed by the *PAB1-K131R-GFP* unacetylated mimic. Previous studies have identified roles for KATs Gcn5 and NuA4 and KDACs Rpd3, Hst1, and Sir2 in SG dynamics (11, 12). Hence, here we opted to directly screen KAT mutants *gcn5Δ* (ADA and SAGA complexes)*, eaf1Δ, eaf7Δ* (NuA4 complex), KDAC mutants *rpd3Δ* (Rpd3S and Rpd3L complexes), *hos3Δ,* and Sirtuin family members *sir2Δhst1Δhst2Δ* (33). Due to functional redundancies, we also opted to screen *sas2Δsas3Δ* (SAS and NuA3 complex). Mutants were transformed with plasmids expressing *PAB1-GFP*, *PAB1-K131Q-GFP*, or *PAB1-K131R-GFP*, and the glucose-deprived SG formation was measured. As previously shown, several KATs/KDACs are involved in the formation of glucose-deprived SGs (11, 12). However, only the KDAC mutant *rpd3Δ* displayed the anticipated phenotypes of decreased glucose-deprived SG formation that was fully suppressed by *PAB1-K131R-GFP* and was epistatic with *PAB1-K131Q-GFP* (Fig. 4).

### Glucose-deprived SG defects of *rpd3Δ* cells can be partially suppressed by *PAB1-K131R-GFP*

**Figure 5:**
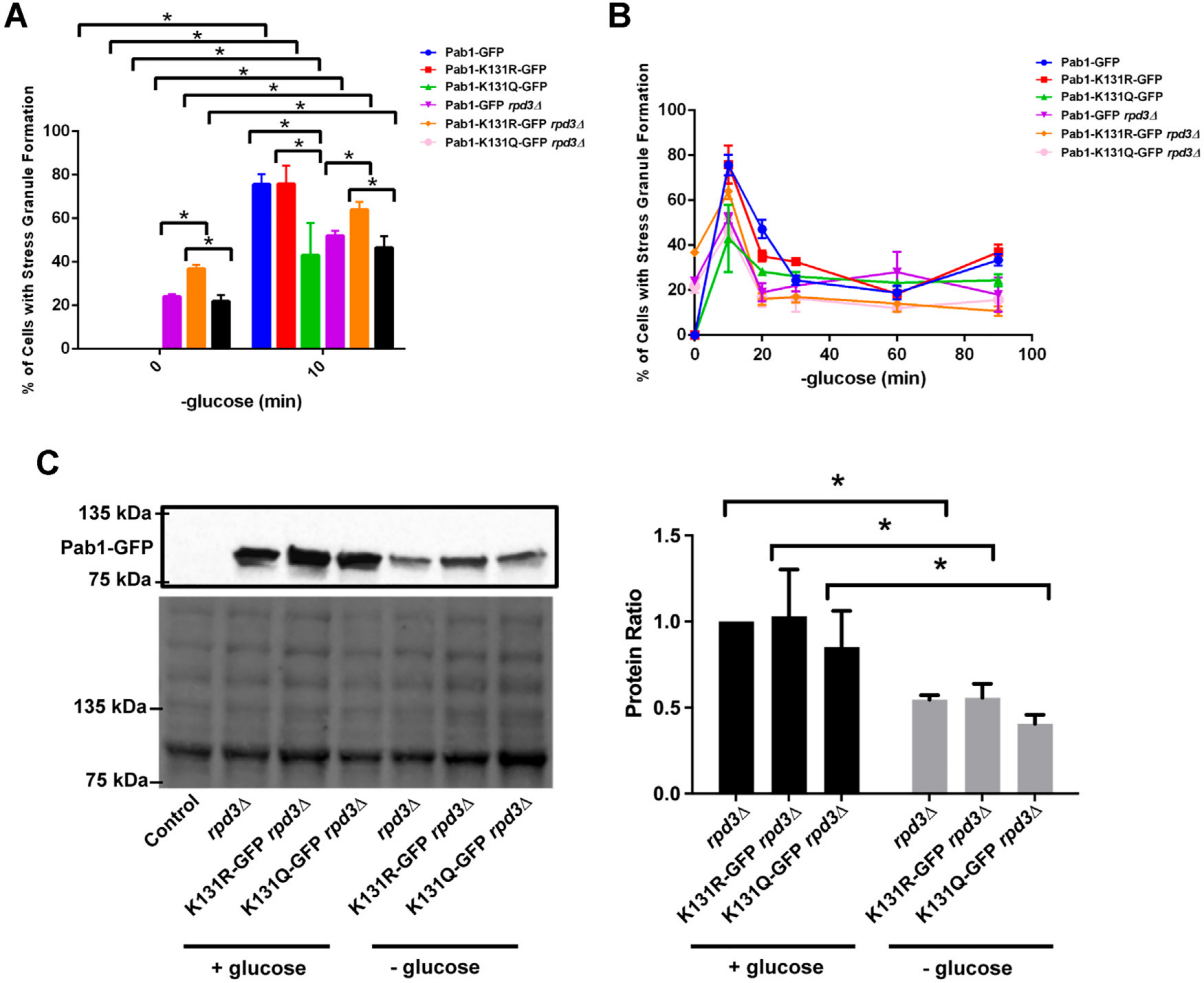
***PAB1-K131R-GFP* partially suppresses glucose-deprived stress granule defects of *rpd3Δ* cells.***A*, quantitative analyses on the stress granule formation of 0-, 10-min of glucose deprivation in *PAB1-GFP* (YKB3114), *PAB1-K131R-GFP* (YKB4395), *PAB1-K131Q-GFP* (YKB4396), *rpd3Δ PAB1-GFP* (YKB5142), *rpd3Δ PAB1-K131R-GFP* (YKB5143), and *rpd3Δ PAB1-K131Q-GFP* (YKB5144). *B*, quantitative analyses on the stress granule formation at 0-, 10-, 20-, 30-, 60-, and 90-min of glucose deprivation in the strains indicated previously. For *A* and *B*, results are the average of three biological replicates, a minimum of 100 cells per replicate were scored, and the error bars indicate SEM. ∗*p* < 0.05 determined using a two-way ANOVA test. *C*, Pab1-GFP protein levels were assessed by quantitative Western blot analysis using whole cell extracts from the indicated strains expressing the indicated *PAB1-GFP* mutant in *rpd3Δ* cells. *Left panel* is representative of anti-GFP Western and total protein blot; *right panel* shows the quantification of Western blots for three independent biological replicates where the Pab1-GFP band intensity was normalized to total protein. ∗*p* < 0.05 determined using a two-way ANOVA test. + Glucose, media containing glucose; - Glucose, media without glucose; Pab1, poly(A)-binding protein 1.

Since we observed decreased glucose-deprived SG formation in *rpd3Δ* cells that was suppressed by plasmid expressed *PAB1-K131R-GFP*, we next sought to determine if this phenotype is replicated when the mutants are expressed from their endogenous genomic locations. Similar to the plasmid-based assay and previous studies (11), *rpd3Δ* cells expressing endogenous *PAB1-GFP* have reduced rate of glucose-deprived SG formation (Fig. 5*A*). *rpd3Δ* cells expressing endogenous *PAB1-K131R-GFP* had an elevated rate of glucose-deprived SGs, but *rpd3Δ* cells expressing endogenous *PAB1-K131Q-GFP* form glucose-deprived SG in similar levels to *rpd3Δ* cells expressing *PAB1-GFP* (Fig. 5*A*). The reduction of glucose-deprived SG formation in *rpd3Δ* cells expressing *PAB1-GFP* or *PAB1-K131Q-GFP* was not reflective of a delay in SG formation (Fig. 5*B*). To assess if changes in SG formation simply reflect changes in protein levels, quantitative Western blot analysis was performed and Pab1-GFP levels were significantly reduced in *rpd3Δ* independent of the mutation (Fig. 5*C*). This suggests that decreases in Pab1-GFP protein levels in *rpd3Δ* upon glucose deprivation may contribute to the decrease of glucose-deprived SG formation in the *rpd3Δ* background (Fig. 5*C*); however, protein levels do not contribute to the ability of *PAB1-K131R-GFP* to suppress defects in glucose-deprived SG formation in *rpd3Δ* cells. Together, our work suggests that Rpd3 deacetylation of Pab1-K131 is required for full SG formation upon glucose deprivation.

### Pab1 RRM1-RRM2-K131Q reduces the binding to poly(A) mRNA

**Figure 6:**
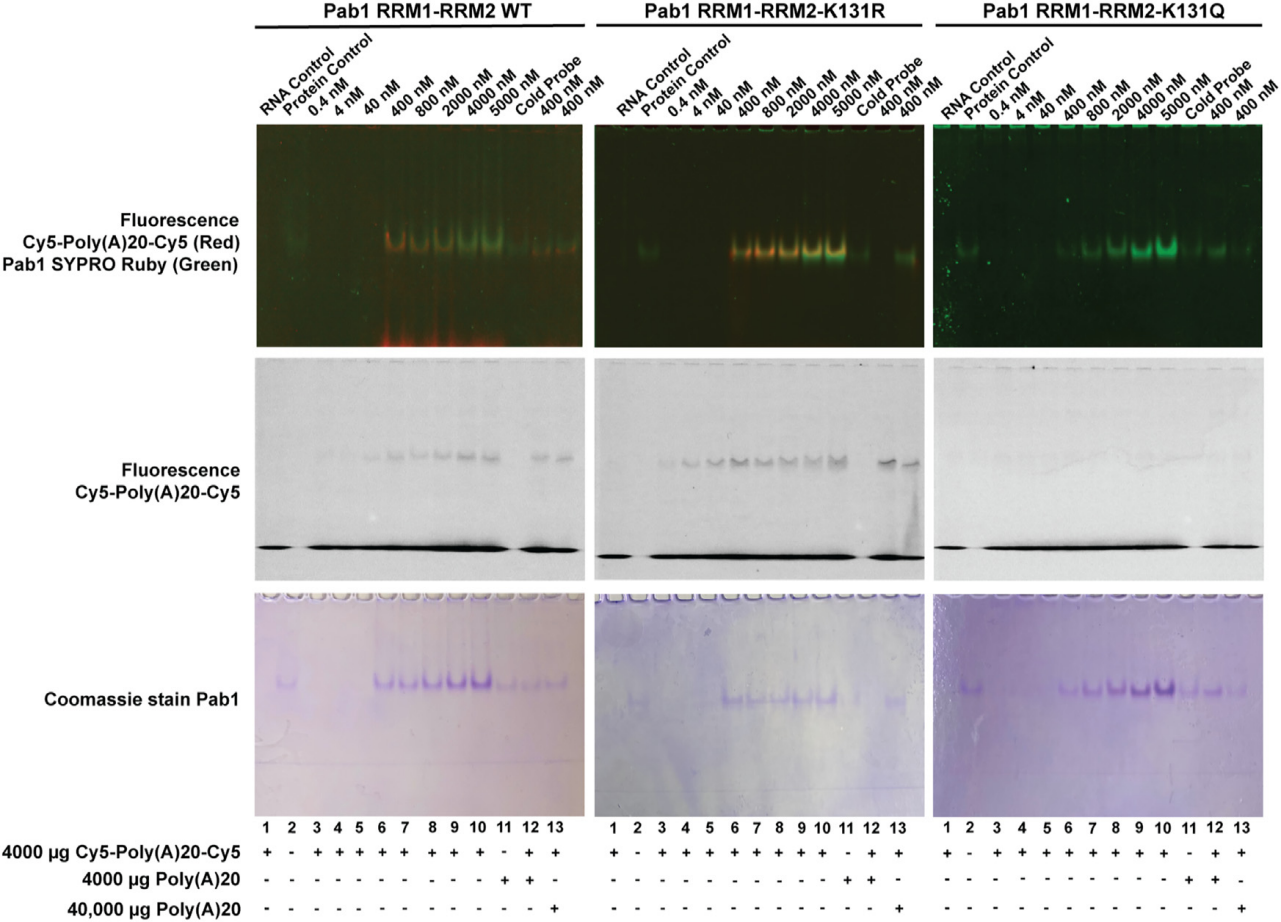
**Pab1 RRM1-RRM2-K131Q reduces binding to poly(A) mRNA.** EMSAs were performed by incubating purified Pab1 RRM1-RRM2 (pKB349), Pab1 RRM1-RRM2-K131R (pKB350), or Pab1 RRM1-RRM2-K131Q (pKB351) at concentrations of 0.4, 4, 40, 400, 800, 2000, 4000, and 5000 nM with Cy5-Poly(A)20-Cy5 mRNA in a binding reaction for 30 min at 30 °C. Pab1 RRM1-RRM2 (*left column*), Pab1 RRM1-RRM2-K131R (*center*), and Pab1 RRM1-RRM2-K131Q (*right column*). For each EMSA, lane 1 is the Cy5-Poly(A)20-Cy5 control, lane 2 is the respective Pab1 protein control (400 ng), lanes 3 to 10 represent the titration of protein with Cy5-Poly(A)20-Cy5, lane 11 is cold probe (Poly(A)20 *control*), lane 12 contains 1:1 ratio between Cy5-Poly(A)20-Cy5 and cold probe, and lane 13 contains 1:10 ratio between Cy5-Poly(A)20-Cy5 and cold probe. *Top row* illustrates a multichannel fluorescent image containing Cy5-Poly(A)20 signal (*red*) and Pab1 stained with SYPRO Ruby signal (*green*). *Center row* is a single channel fluorescent image of Cy5-Poly(A)20. *Bottom row* corresponds to the Coomassie stain of the gels showing Pab1 RRM1-RRM2, Pab1 RRM1-RRM2-K131R, and Pab1 RRM1-RRM2-K131Q. Image presented is representative of three replicates. Pab1, poly(A)-binding protein 1; RRM, RNA recognition motif.

We next sought to explore the mechanism(s) by which acetylation of Pab1-K131 might impact SG formation. As K131 is in the RRM2 portion of Pab1 implicated in interactions with transcription initiation factors and RNA, we explore these two possibilities. First, we asked if Pab1-K131 influenced its interaction with transcription initiation factor eIF4G1 (11), and we determined this was not the case (Fig. S1). As RRM2 is also responsible for anchoring and stabilization of poly(A) RNA tail, we next sought to determine if acetylation of Pab1-K131 might cause a change in RNA-binding affinity, affecting glucose-deprived SG formation. Previous EMSA studies have shown that incubation of poly(A) RNA tail tagged with Cy5 with Pab1 RRM1-RRM2 protein fragment results in a shift of the Cy5-Poly(A)20-Cy5 band, which can be competed away upon coincubation with increasing amounts of untagged poly(A)20 (29). Hence, we employed EMSA to elucidate if K131 acetylation and nonacetylation mimics fragments of RRM1-RRM2 impact its interaction with poly(A) RNA. Pab1 RRM1-RMM2-K131R binds Cy5-Poly(A)20-Cy5 in a similar fashion to Pab1 RRM1-RMM2 protein fragment, with a slight increase in affinity presented by the nonacetylated mimic (Figs. 6 and S2). In contrast, Pab1 RRM1-RRM2-K131Q binding to Cy5-Poly(A)20-Cy5 is dramatically reduced. This suggests that acetylation at Pab1-K131 reduces its ability of Pab1 RRM1-RRM2 fragment to bind onto poly(A) mRNA, which can affect redirecting and further protection of mRNA from degradation into SG.

## Discussion

Here, we show that yeast expressing the acetylation mimic *PAB1-K131Q-GFP* selectively decreases SG formation upon glucose deprivation, ethanol, raffinose, and vanillin (Fig. 3). However, the acetylation mimic does not globally decrease SG formation as it has no discernable impact of SG formation upon heat shock and glycerol. Our genetics reconfirm that although several KAT and KDAC enzymes are implicated in SG dynamics, Rpd3 maybe the KDAC responsible for deacetylation of Pab1-K131 upon glucose deprivation (Figs. 4 and 5). Deletion of *RPRD3* displayed the anticipated phenotypes of decreased glucose-deprived SG formation, which was fully suppressed by *PAB1-K131R-GFP* and was epistatic with *PAB1-K131Q-GFP.* Further, our biochemistry suggests that acetylation of Pab1-K131 in the RRM2 domain may reduce interaction with poly(A) mRNA (Fig. 6). We proposed a model where Pab1-K131 maybe acetylated under nonstress conditions, inhibiting interaction with poly(A) RNA and SG formation. Upon glucose deprivation, Pab1-K131 is deacetylated by Rpd3 which allows for increased interaction with poly(A) mRNA and for increase in SG formation. Pab1-K131Q-GFP can still form glucose-deprived SGs, albeit at a lower rate, which suggests this is just one signaling pathway contributing to glucose-deprived SG formation.

To our knowledge, Pab1-K131 is the first acetylation site directly implicated in SG formation in yeast. However, numerous studies in mammalian cells have shown that acetylation and deacetylation of a variety of proteins influence SGs, including acetylation of RNA helicase DDX3X, FUS, and G3BP1 (34, 35, 36). This suggests that acetylation maybe a conserved key signaling pathway, regulating SG dynamics. In agreement with this hypothesis, many yeast core SG-localized proteins such as Pub1 and Pbp1 are also highly acetylated (17, 37, 38). Indeed, acetylation maybe an ideal signaling pathway to modulate SG formation under distinct stresses. As shown here, Pab1-K131Q-GFP only impacts SG formation upon ethanol, glucose deprivation, raffinose, and vanillin, but not heat shock or glycerol (Fig. 3). One possibility is that lysine acetylation sites and/or proteins that are acetylated change upon stresses. Having an “acetylation-code” may contribute to distinct SG compositions under different stresses, as lysine acetylation has an established role in regulating protein–protein or protein-DNA/RNA interactions (39, 40, 41). For example, Pab1 domains required for SG formation under heat shock and glucose deprivation are different. While glucose-deprived SGs rely on RRM1 and RRM2 domains of Pab1, heat shock SGs formed rely on the RRM3 domain of Pab1 (42). This may also explain why so many KATs and KDACs have been implicated in various aspects of SG dynamics. Moving forward, to truly dissect out the individual role of specific acetylation sites and KATs/KDACs will require detailed quantitative acetylome studies to identify acetylation sites on SG-associated proteins that change upon individual stresses and their dependence on individual KAT and KDACs. Building a global and temporal SG acetylome map will be the first step towards a holistic understanding of how acetylation is regulating SG dynamics.

Deletion mutants of Rpd3 displayed decreased SG formation upon glucose deprivation that was suppressed by *PAB1-K131R-GFP* and was epistatic with *PAB1-K131Q-GFP* (Fig. 5, *A* and *B*). Further, Rpd3 maybe involved in Pab1 protein level regulation. Though *PAB1-K131Q-GFP* and *PAB1-K131R-GFP* did not impact protein levels in cells expressing Rpd3 (Fig. 5*C*), upon glucose depletion, Pab1-GFP proteins levels were significantly reduced in *rpd3Δ* cells, independent of K131 mutation. Given Rpd3’s established role in transcription (43, 44, 45), a likely scenario is that Rpd3 regulates the transcription of Pab1 under this stress. Regardless of the mechanism, the decrease in Pab1-GFP protein levels in *rpd3Δ* cells cannot fully explain the reduction of glucose-deprived SG formation, as the nonacetylated mimic still presents more granule formation despite having a similar reduction in protein levels (Fig. 5*C*). While we know that the Pab1-K131 acetylation state may also contribute to SG formation under ethanol, raffinose, and vanillin, we do not know if Rpd3 is also implicated in regulating SG formation for these stresses. Further, as *rpd3Δ* cells have elevated SG formation even under glucose replete conditions (Fig. 5*A* and (11)) that is not suppressed by Pab1-K131R-GFP, it suggests that Rpd3 may have other targets, regulating SG dynamics beyond just Pab1-K131.

What is the KAT required for Pab1-K131 acetylation? We anticipated that the deletion mutant of the KAT(s) responsible for Pab1-K131 acetylation would have increased SGs that would be suppressed by the acetylation mimic. However, none of the KAT mutants screened displayed this phenotype, including *eaf1Δ* and *gcn5Δ* (Fig. 4), which have both been implicated in the formation of SG under glucose deprivation (11). Interestingly, in contrast to the results in *rpd3Δ* cells, both *PAB1-K131Q-GFP* and *PAB1-K131R-GFP* had the same impact in *gcn5Δ* cells, a significant reduction in glucose-deprived SG formation (Fig. 4). This suggests that in the context of *GCN5* deletion background, the acetylation status of Pab1-K131 might not be as important as the lysine amino acid itself. Though not significant, a similar trend was seen in *eaf1Δ* and *eaf7Δ* cells. In contrast, Pab1-K131R-GFP partially rescued the glucose-deprived SG formation defects in *sas2Δsas3Δ* cells (Fig. 4). As Pab1-K131R-GFP is not an acetylation mimic, the rescue is not because Sas2 or Sas3 acetylate Pab1-K131, in which case we anticipate that Pab1-K131Q-GFP would rescue. Rather, it suggests that Pab1-K131R-GFP is suppressing the *sas2Δsas2Δ* defects through a parallel mechanism. Given that KATs, like KDACs, might have multiple target sites on multiple SG-associated proteins which fine-tune SG formation, it may be a challenge to identify the specific KAT required for Pab1-K131 acetylation through genetic approaches shown here. To find the KAT(s) responsible for a specific acetylation site may require quantitative acetylome studies and/or *in vitro* biochemical approaches coupled to MS (18). Alternatively, it may be possible that these sites are acetylated through chemical or nonenzymatic acetylation. Although *in vitro* studies have shown the possibility of nonenzymatic lysine acetylation, this mechanism is highly dependent on cell pH levels, with the mitochondrial compartment presenting a pH that is permissive of nonenzymatic acetylation, whereas the cytoplasmic and nuclear compartments being less permissive (46). Nonetheless, further studies should be conducted to determine if Pab1-K131 is susceptible to enzyme-independent acetylation.

Though multiple groups have detected acetylation at Pab1-K131, we cannot rule out the possibility that the Pab1-K131R and Pab1-K131Q might not fully replicate the unacetylated or acetylated state, respectively. Regardless, our work suggests that either acetylation of Pab1-K131 or amino acid at position 131 impacts glucose-deprived SG formation. Pab1-K131 is within the RRM2 domain of the protein, which has a role in translation by direct interaction with the poly(A) RNA tail (47, 48). Previous mutational scans have shown that Pab1-K131 residue is sensitive to almost all amino acid substitutions (49). Molecular structure predictions using SIFT (https://sift.bii.a-star.edu.sg/) (50) showed that any mutation on K131 is unfavorable, reinforcing that any changes cause a significant impact on protein–RNA interactions. As such, even posttranslational modifications such as residue acetylation might disrupt protein–RNA interactions, leading to a failure to form aggregates into SGs and SG formation itself. Indeed, acetylation of RRM–RNA-binding domains may be a common mechanism to disrupt RNA binding and influencing SG formation. The RAS GTPase-activating protein-binding protein G3BP1 is critical for SG formation in mammalian cells, and acetylation of K376 of its RRM domain impairs its RNA binding and SG assembly (36). Though the alignment between the RRM domain of G3BP1 and RRM2 of Pab1 is relatively low, 3GBP1-K376 site aligns with Pab1-K164, a site that we were not able to successfully assess. Future studies will be needed to assess the impact of Pab1-K164 acetylation on SG formation and RNA binding.

**Figure 7:**
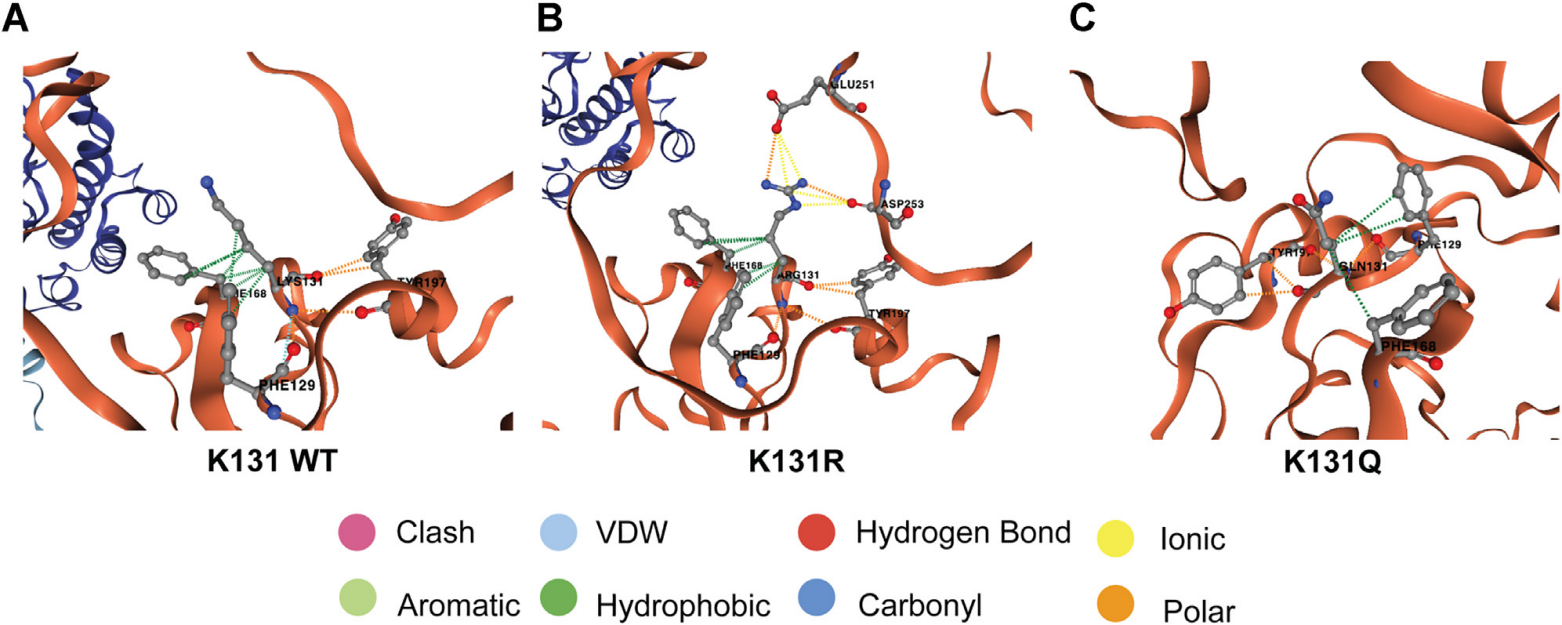
**Predicted residue interactions show changes in nonacetylated and acetylated mimic behaviors.** Dynamut2 predictions of Pab1 RRM1-RRM2 WT (*A*) and nonacetylated mimic R (*B*) show hydrophobic interactions between the aromatic portions of Tyr197, Phe129, and 168, which is believed to stabilize the protein structure. Polar interactions between K131R and Glu251 and Asp253 were predicted and are also related to structural stability. In the acetylated mimic Q (*C*), the number of overall predicted interactions is smaller than the WT and nonacetylated mimic, with a significant decrease of hydrophobic interactions between the residue and Phe168, which could be linked to a decrease of structural stability. Pab1, poly(A)-binding protein 1; RRM, RNA recognition motif; VDW, van der Waals force.

Several studies have shed light on the importance of hydrogen bonding in nucleic acid base recognition and binding specificity, with emphasis on donor−acceptor shape and locations on each RNA nucleobase (51). Based on these trends, we predicted that the nonacetylated mimic (R) would have stronger binding affinity than the acetylated mimic (Q), due to RNA negative electrostatic phosphate backbone’s likeliness to interact with positively charged residues (52). Indeed, electromobility shift assays showed that Pab1 RRM1-RRM2-K131Q has a reduced ability to bind onto poly(A) mRNA, whereas the nonacetylated mimic presented higher binding levels to the unmodified protein (Fig. S2). Hydrogen-bonding properties of amino acids and RNA bases analysis showed that lysine is more prone to bind to RNA while uracil, followed by adenine, have the highest propensity to bind to protein (53). Dynamut 2 (54) and Missense3D predictions (55) demonstrated that the Pab1 nonacetylated mimic breaks the original salt bridge bond between K131 to D208 but creates salt bridges between R131 and E251 and D253. It also creates multiple hydrogen bonds: R131-E206, R131-D208, and R131 E251 around NH1, and R131-E206 and R131-D208 around NH2, whereas the acetylated mimic breaks the original salt bridge bond, and no hydrogen bonds are formed (Figs. 7 and S3).

Although the WT and the nonacetylated mimic possess a positive charge at residue 131, in the WT, it is due to its lysine amino group, while the mimic has a guanidinium group which is unique to arginine. These groups respond differently to hydrogen binding, where the amino groups form monodentate bonds, while guanidinium groups form bidentate bonds (56). Polydentate hydrogen bonds tend to be more stable than monodentate ones, conferring higher stability to the RNA–protein binding complex between the arginine guanidinium group and the phosphate groups of the poly(A) mRNA. Prediction of structural arrangements using optimized models show stable hydrogen bond interactions between the arginine guanidinium moiety and adenine (57). This throws light on the increase in binding affinity for the nonacetylated mimic in comparison to the acetylated mimic and WT Pab1, respectively, in addition to the potential creation of new salt bridges and hydrogen bonds.

In conclusion, by using yeast as a model organism and mimicking the acetylation site of Pab1-K131, we showed a significant decrease in the SGs formed under glucose deprivation. The KDAC enzyme Rpd3 was shown to be a potential part of this process by affecting the Pab1 cellular protein levels. Moreover, acetylation mimic of Pab1-K131 reduces its ability to bind onto RNA, showing that acetylation of this lysine residue is pivotal in the regulation of SG dynamics.

## Experimental procedures

### Yeast strains, plasmids, and media

Strains and plasmids used in this study are listed in Tables S1 and S2. Plasmids and strains were created by the modification of chromosomal genes through PCR and homologous recombination (58). *PAB1-K131R-GFP* and *PAB1-K131Q-GFP* were generated at their endogenous genomic locations *via* CRISPR/Cas9 (59). Yeast cultures were grown at 30 °C at 200 rpm in either Yeast Peptone Dextrose (YPD) medium (w/v 1% yeast extract, 2% peptone, and 2% dextrose), Yeast Peptone medium (w/v 1% yeast extract, 2% peptone), Synthetic Complete Dextrose (SCD) medium (w/v 0.67% yeast nitrogenous base without amino acids, 0.2% amino acid dropout mix, 2% dextrose), or Synthetic Complete medium (w/v 0.67% yeast nitrogenous base without amino acids, 0.2% amino acid dropout mix).

### SG assessment

Yeast transformed with GFP-tagged plasmids were grown in SCD-Uracil media, and GFP-tagged integrated mutants were grown in YPD media. For glucose deprivation, cultures were grown in YPD or SCD-Uracil media until mid-log phase (*A*600 ∼ 0.5–0.8) and centrifuged at 3000 rpm for 3 min at room temperature prior to being resuspended in the same glucose-deprived media. Cells were grown at 30 °C and harvested for analysis following experiment-specific incubation times. For 20 nM ethanol, 3% glycerol, 2% raffinose, and 30 nM vanillin stressors, cells were collected as for glucose deprivation. However, cells under 20 nM ethanol, 3% glycerol, and 2% raffinose stress were resuspended in Yeast Peptone media with the additional carbon sources, whereas cells under 30 nM vanillin stress were resuspended in YPD media. Cells were grown at 30 °C for 30 min and harvested for analysis. For temperature stress, cells were collected as for glucose deprivation, resuspended in YPD media preconditioned to 46 °C, and grown at 46 °C for 30 min prior to being harvested for analysis.

### Fluorescence microscopy

Microscopy was completed using a Leica DMI 6000 microscope equipped with a Hamamatsu Orca AG camera, a Sutter DG4 light source, a Ludl emission filters wheel with Chroma bandpass emission filters (Leica Microsystems GmbH). For each frame, Z-stacked images (0.2 μm across 7 μm) were obtained using Volocity 4.3.2 (PerkinElmer) using a 63× objective without binning and 3 s exposure for GFP fluorescence. Images were analyzed using NIH FIJI ImageJ software (https://imagej.nih.gov/ij/download.html) (60).

Microscopy experiments were completed in triplicate, and each replicate consisted of at least 100 cells. The number of total cells and number of cells that form SGs were determined by manual counting of stacked images. From these values, the percentage of cells that form SGs was determined. Statistics was completed using an online *t* test calculator by GraphPad Prism (https://www.graphpad.com/quickcalcs/ttest1.cfm). ANOVA was utilized, with a *p*-value of <0.05 representing statistical significance.

### Dot assays

Cultures were grown to mid-log in YPD before being diluted to an *A*600 0.1. Three 10-fold serial dilutions (0.01, 0.001, and 0.0001) of 5 μl of the serial dilutions were spotted onto the indicated plates. The plates were incubated at 30 °C for 24 h. Images of dot assays were taken using the Bio-Rad ChemiDoc XRS system under epi-white light illumination and Image Lab software version 5.2 (Bio-Rad Laboratories, Inc).

### Pab1 RRM1-RRM2 fragment expression and purification

The DNA encoding Pab1 RRM1-RRM2 fragment, Pab1 RRM1-RRM2-K131Q, and Pab1 RRM1-RRM2-K131 R were synthesized by GenScript (GenScript Biotech Corporation) and codon optimized for expression in *Escherichia* coli. Fragments were cloned into EcoRI and Notl restriction sites of the pGEX-4T-1 plasmid and all plasmids were PCR confirmed. The constructs were transformed into chemically competent BL21 *E. coli* cells for protein expression and extraction. Bacterial cultures were induced with 0.25 mM IPTG for 2 h at 37 °C. The culture was spun down at 3000 rpm for 3 min, washed with 1 x PBS, and spun down again prior to weighting the pellet. The cells were resuspended with 2.5 ml/g of freezing buffer (1xPBS, 1 mM EDTA, 1 mM EGTA, and 1 mM PMSF) and froze down at -80 °C. Samples were thawed and 2.5 ml/g of freezing buffer supplemented with 15 mM DTT. To this, 0.5% Triton X-100 was added, followed by sonication (Misonix Sonicator 3000 550W, Cole-Parmer Instrument Company) four times for 30 s, with 1 min resting period on ice. The samples were spun down at 4000 rpm for 20 min, and the supernatant was collected. Glutathione Sepharose 4b (Life Technologies) was washed in washing buffer (1× PBS, 0.1% NP-40, 0.5 M NaCl, 1 mM DTT, 1 mM EDTA, 1 mM EGTA, and 1 mM PMSF). Five hundred microliters of glutathione sepharose and the samples’ supernatants were batch bonded in a closed column for 2 h at 4 °C on an end-over-end rotation. The flow-through was collected. The column was washed with 10 ml of washing buffer and 2 ml of cleavage buffer (1× PBS, 140 mM Na_2_HPO_4_, 1.8 mM KHPO, 138 mM NaCl, and 2.7 mM KCl pH 7.2). One microliter of PBS and thrombin proteases was added to a closed column and rotated overnight at room temperature. The eluate was drained and washed with 2 ml of cleavage buffer. The fragments were diluted in exchange protein buffer (20 mM Tris–HCl (pH 7.4), 100 mM KOAc, 2 mM MgOAc, 1 mM PMSF, and 10% glycerol) and concentrated using a 10K 20 ml concentrator, following a standard protocol (Thermo Fisher Scientific).

### EMSA of Pab1 fragments

The interaction between Pab1 and polyA20 was assessed by using EMSA. The RNAs used in this study were generated from hydrolyzed Cy5-PolyA20-Cy5 and PolyA20 (Sigma-Aldrich). Protein fragments were diluted in a protein buffer (20 mM Tris–HCl pH 7.4, 100 mM KOAc, 2 mM MgOAc, and 10% glycerol) to a final concentration of 0.2 μg/μl. Binding reactions (20 μl) were incubated in 4 μl of 5× binding buffer (50 mM Tris–HCl (pH 7.5), 750 mM KCl, 0.5 mM EDTA, and 0.5 mM DTT), protein buffer, 4 μl of RNA, and 0.4 nM, 4 nM, 40 nM, 400 nM, 800 nM, 2000 nM, 4000 nM, or 5000 nM of either Pab1 RRM1-RRM2, Pab1-K131R RRM1-RRM2, or Pab1-K131Q RRM1-RRM2 with its respective concentration at 30 °C for 30 min. 1.5 mm thick 10% nondenaturing PAGE (37.5:1, w/w) was prerun for 30 min at 20 V in 0.4× TBE buffer. Twenty microliters of samples were loaded with the gel still running. The voltage was changed to 55 V, and samples were run in a 4 °C cold room with the gel chamber submerged in a bucket of ice for 5 h. Gels were imaged using the Bio-Rad ChemiDoc XRS system under Cy5 and SYPRO Ruby multichannel mode, and pictures were merged using Image Lab Software version 5.2 (Bio-Rad Laboratories). Relative RNA binding was quantified using Image Lab, with each band normalized to the relative control RNA band.

### Statistical analysis

Statistics was performed using GraphPad Prism. When comparing two groups, a Student’s *t* test was performed, and in more than two groups analysis, two-way ANOVA was performed. In all analyses, *p* < 0.05 to define significance was used.

## Data availability

All data is contained within the article.

## Supporting information

This article contains supporting information.

## Conflict of interest

The authors declare that they have no conflicts of interest with the contents of this article.
