# Supplementary material for: Pab1 acetylation at K131 decreases stress granule formation in Saccharomyces cerevisiae
Source: J Biol Chem. 2022 Dec 24;299(2):102834. doi: 10.1016/j.jbc.2022.102834 (PMC9867979; doi:10.1016/j.jbc.2022.102834)
Supplement: Supporting information [file mmc1.pdf]

Pab1-K131 acetylation effects in stress granules

Supporting information

Table S1: strains used in this study

Strain	Genotype
**YKB1079**	*MATa his3Δ1 leu2Δ0 met15Δ0 ura3Δ0*
**YKB3114**	*MATa his3Δ1 leu2Δ0 met15Δ0 ura3Δ0 [pPab1-GFP::HIS]*
**YKB4033**	*MATa his3Δ1 leu2Δ0 met15Δ0 ura3Δ0 pab1ΔKANMX [pPab1-GFP::URA]*
**YKB4035**	*MATa his3Δ1 leu2Δ0 met15Δ0 ura3Δ0 pab1Δ::KANMX[pPab1-K131R-GFP::URA]*
**YKB4036**	*MATa his3Δ1 leu2Δ0 met15Δ0 ura3Δ0 pab1Δ::KANMX[pPab1-K131Q-GFP::URA]*
**YKB4037**	*MATa his3Δ1 leu2Δ0 met15Δ0 ura3Δ0 pab1Δ::KANMX[pPab1-K7R-GFP::URA]*
**YKB4038**	*MATa his3Δ1 leu2Δ0 met15Δ0 ura3Δ0 pab1Δ::KANMX [pPab1-K7Q-GFP::URA]*
**YKB4039**	*MATa his3Δ1 leu2Δ0 met15Δ0 ura3Δ0 pab1Δ::KANMX [pPab1-K288R-GFP::URA]*
**YKB4040**	*MATa his3Δ1 leu2Δ0 met15Δ0 ura3Δ0 pab1Δ::KANMX [pPab1-K288R-GFP::URA]*
**YKB4041**	*MATa his3Δ1 leu2Δ0 met15Δ0 ura3Δ0 pab1Δ::KANMX [pPab1-K504R-GFP::URA]*
**YKB4042**	*MATa his3Δ1 leu2Δ0 met15Δ0 ura3Δ0 pab1Δ::KANMX [pPab1-K504Q-GFP::URA]*
**YKB4395**	*MATa his3Δ1 leu2Δ0 met15Δ0 ura3Δ0 Pab1-K131R-GFP::HIS [pRS415-TEF1p-Cas9-CYC1t] [p426-SNR52p-gRNA. PAB1 (PAM seq #33) - SUP4t]*
**YKB4396**	*MATa his3Δ1 leu2Δ0 met15Δ0 ura3Δ0 Pab1-K131Q-GFP::HIS [pRS415-TEF1p-Cas9-CYC1t] [p426-SNR52p-gRNA. PAB1 (PAM seq #33) - SUP4t]*
**YKB4969**	*MATa his3Δ1 leu2Δ0 met15Δ0 ura3Δ0 lys2Δ0 eaf7Δ::NATMX [pPab1-GFP::HIS]*
**YKB4970**	*MATa his3Δ1 leu2Δ0 met15Δ0 ura3Δ0 lys2Δ0 eaf7Δ::NATMX [pPab1-K131R-GFP::HIS]*
**YKB4971**	*MATa his3Δ1 leu2Δ0 met15Δ0 ura3Δ0 lys2Δ0 eaf7Δ::NATMX [pPab1-K131Q-GFP::HIS]*
**YKB4175**	*MATa his3Δ1 leu2Δ0 met15Δ0 ura3Δ0 eaf1Δ::NATMX [pPab1-GFP::HIS]*
**YKB4965**	*MATa his3Δ1 leu2Δ0 met15Δ0 ura3Δ0 lys2Δ0 eaf1Δ::NATMX [pPab1-K131R-GFP::HIS]*
**YKB4964**	*MATa his3Δ1 leu2Δ0 met15Δ0 ura3Δ0 lys2Δ0 eaf1Δ::NATMX [pPab1-K131Q-GFP::HIS*
**YKB4177**	*MATa his3Δ1 leu2Δ0 met15Δ0 ura3Δ0 gcn5Δ::NATMX [pPab1-GFP::HIS]*
**YKB4178**	*MATa his3Δ1 leu2Δ0 met15Δ0 ura3Δ0 gcn5Δ::NATMX [pPab1-K131R-GFP::HIS]*
**YKB4179**	*MATa his3Δ1 leu2Δ0 met15Δ0 ura3Δ0 gcn5Δ::NATMX [pPab1-K131Q-GFP::HIS]*
**YKB4180**	*MATa his3Δ1 leu2Δ0 met15Δ0 ura3Δ0 rpd3Δ::KANMX [pPab1-GFP::HIS]*
**YKB4181**	*MATa his3Δ1 leu2Δ0 met15Δ0 ura3Δ0 rpd3Δ::KANMX [pPab1-K131R-GFP::HIS]*
**YKB4182**	*MATa his3Δ1 leu2Δ0 met15Δ0 ura3Δ0 rpd3Δ::KANMX [pPab1-K131Q-GFP::HIS]*
**YKB4966**	*MATa his3Δ1 leu2Δ0 met15Δ0 ura3Δ0 lys2Δ0 hos3Δ::KANMX [pPab1-GFP::HIS]*
**YKB4967**	*MATa his3Δ1 leu2Δ0 met15Δ0 ura3Δ0 lys2Δ0 hos3ΔKANMX [pPab1-K131R-GFP::HIS]*
**YKB4968**	*MATa his3Δ1 leu2Δ0 met15Δ0 ura3Δ0 hos3Δ::KANMX [pPab1-K131Q-GFP::HIS]*
**YKB4161**	*MATa his3Δ1 leu2Δ0 met15Δ0 ura3Δ0 lys2Δ0 sas2Δ::KANMX sas3Δ::NATMX [pPab1-GFP::HIS]*
**YKB4162**	*MATa his3Δ1 leu2Δ0 met15Δ0 ura3Δ0 sas2Δ::KANMX sas3Δ::NATMX [pPab1K131R-GFP::HIS]*
**YKB4163**	*MATa his3Δ1 leu2Δ0 met15Δ0 ura3Δ0 sas2Δ::KANMX sas3Δ::NATMX [pPab1-K131Q-GFP::HIS]*
**YKB4166**	*MATa his3Δ1 leu2Δ0 met15Δ0 ura3Δ0 sir2Δ::KANMX hst1Δ::NATMX hst2Δ::HISMX [Pab1-GFP::HIS]*
**YKB4167**	*MATa his3Δ 1leu2Δ0 met15Δ0 ura3Δ0 sir2Δ::KANMX hst1Δ::NATMX hst2Δ::HISMX [pPab1-K131R-GFP::HIS]*

**YKB4168**	*MATa his3Δ1 leu2Δ0 met15Δ0 ura3Δ0 sir2Δ::KANMX hst1Δ::NATMX hst2Δ::HISMX [pPab1-K131Q-GFP::HIS]*
**YKB4703**	*MATa his3Δ1 leu2Δ0 met15Δ0 ura3Δ0 TIF4631-HA::KAN*
**YKB5136**	*MATa his3Δ1 leu2Δ0 met15Δ0 ura3Δ0 Pab1-GFP::HIS eaf1Δ:KANMX*
**YKB5137**	*MATa his3Δ1 leu2Δ0 met15Δ0 ura3Δ0 Pab1-K131R-GFP::HIS eaf1Δ::KANMX*
**YKB5138**	*MATa his3Δ1 leu2Δ0 met15Δ0 ura3Δ0 Pab1-K131Q-GFP::HIS eaf1Δ::KANMX*
**YKB5139**	*MATa his3Δ1 leu2Δ0 met15Δ0 ura3Δ0 Pab1-GFP::HIS gcn5Δ::KANMX*
**YKB5140**	*MATa his3Δ1 leu2Δ0 met15Δ0 ura3Δ0 Pab1-K131R-GFP::HIS gcn5Δ::KANMX*
**YKB5141**	*MATa his3Δ1 leu2Δ0 met15Δ0 ura3Δ0 Pab1-K131Q-GFP::HIS gcn5Δ::KANMX*
**YKB5142**	*MATa his3Δ1 leu2Δ0 met15Δ0 ura3Δ0 Pab1-GFP::HIS rpd3Δ::KANMX*
**YKB5143**	*MATa his3Δ1 leu2Δ0 met15Δ0 ura3Δ0 Pab1-K131R-GFP::HIS rpd3Δ::KANMX*
**YKB5144**	*MATa his3Δ1 leu2Δ0 met15Δ0 ura3Δ0 Pab1-K131Q-GFP::HIS rpd3Δ::KANMX*

Table S2: Plasmids used in this study

**Plasmid**	**Component**
**pKB192**	Pab1-GFP; Cen; *URA3* marker; *[pPAB1-GPF::URA]*
**pKB355**	Pab1-K131R-GFP; Cen; *URA3* marker; *[pPAB1-K131R-GPF::URA]*
**pKB356**	Pab1-K13Q1-GFP; Cen; *URA3* marker; *[pPAB1-K131Q-GPF::URA]*
**pKB349**	pGEX-4T-1-Pab1-RRM1-RRM2-GST
**pKB350**	pGEX-4T-1-Pab1-RRM1-RRM2-K131R-GST
**pKB351**	pGEX-4T-1-Pab1-RRM1-RRM2-K131Q-GST

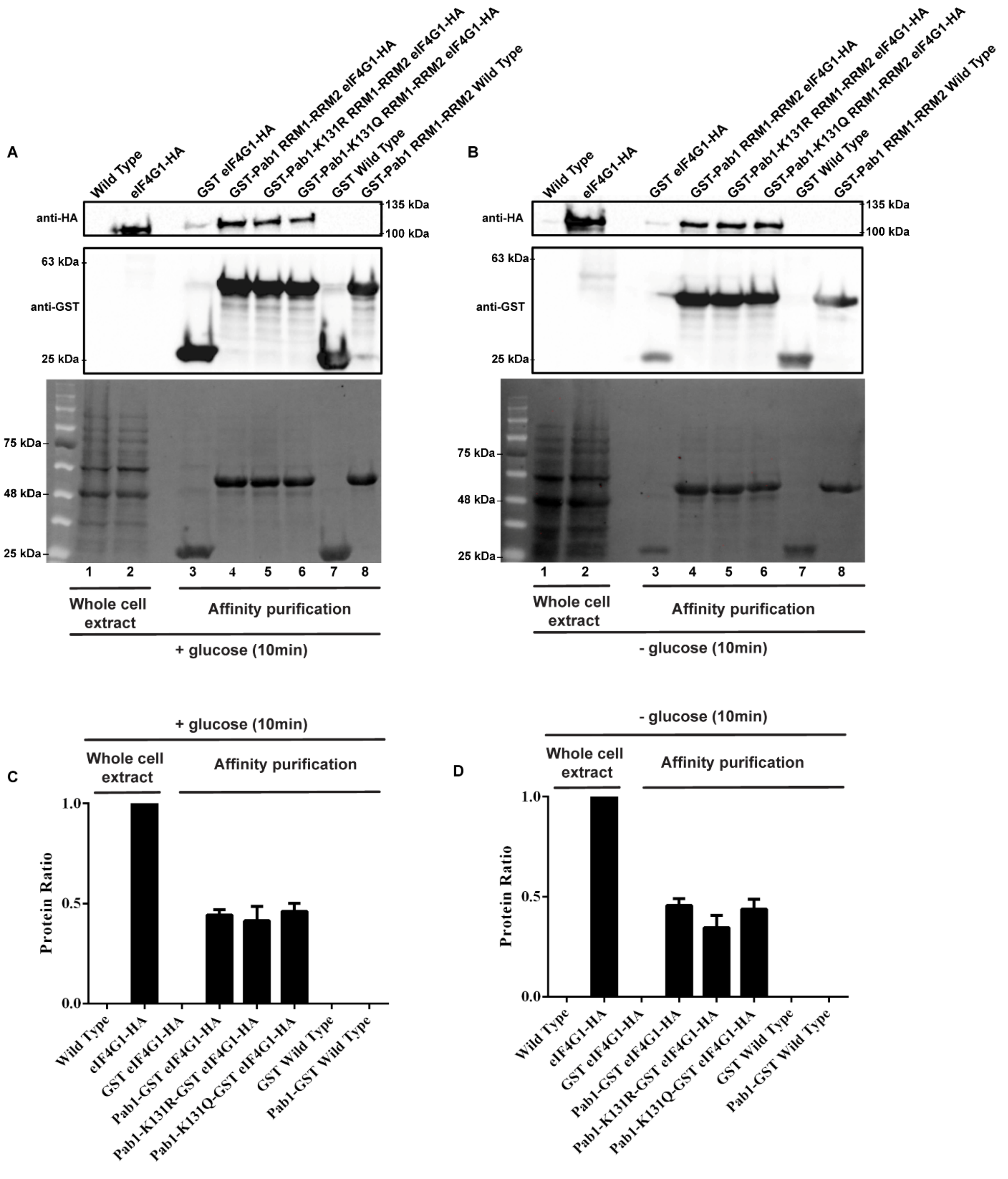

Figure S1: K131 mutants do not impact eIF4G binding upon glucose deprivation. A) Affinity purification analysis performed by incubating GST-Pab1 RRM1-RRM2 (PKB349), GST-Pab1-K131R RRM1-RRM2 (PKB350), and GST-Pab1-K131Q RRM1-RRM2 (PKB351) with eIF4G1. Representative anti-HA western blot (top panel), anti-GST western blot (middle panel), and total protein blot (bottom panel). B) Affinity purification analysis performed by incubating GST-Pab1 RRM1-RRM2 (PKB349), GST-Pab1-K131R RRM1-RRM2 (PKB350), and GST-Pab1-K131Q RRM1-RRM2 (PKB351) with eIF4G1 after 10 minutes of glucose

deprivation. Representative anti- HA western blot (top panel), anti-GST western blot (middle panel), and total protein blot (bottom panel). C) Quantification of western blots for three independent biological replicates, glucose repleted HA band intensity normalized to total protein. D) Quantification of western blots for three independent biological replicates, glucose depleted HA band intensity normalized to total protein. Error bars indicate SEM. Differences between the samples were proven to be insignificant through two-way ANOVA tests.

Pab1-K131 acetylation effects in stress granules

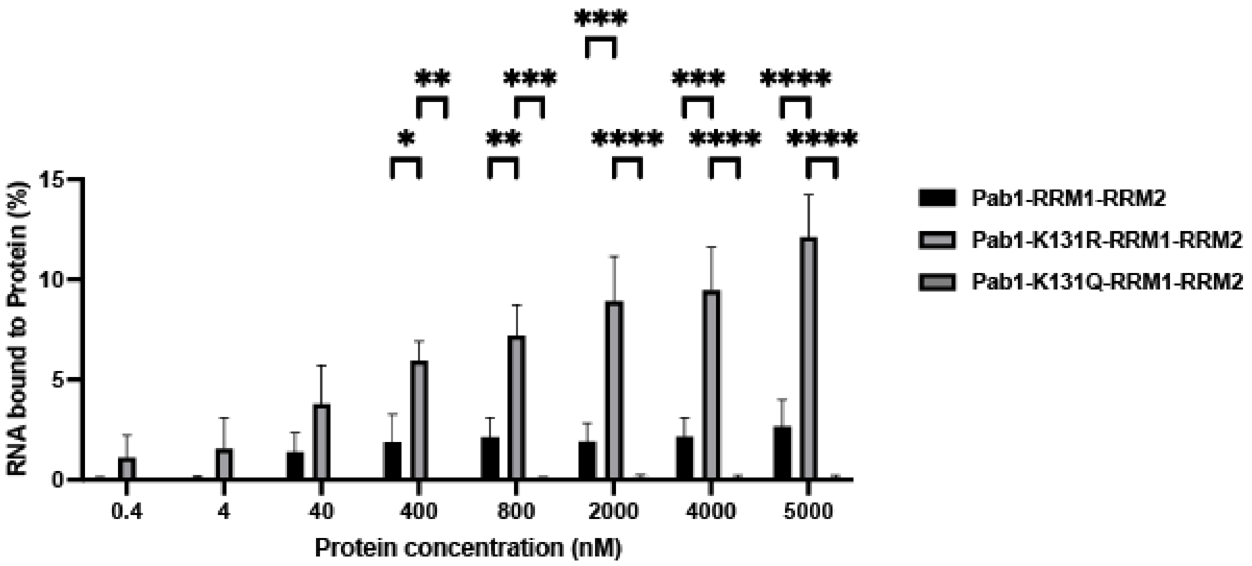

Figure S2: Pab1 acetylated mimic presents higher binding affinity to RNA. Quantification of Electromobility Shift Assay shift bands of Pab1-RRM1-RRM2, Pab1-K131R-RRM1-RRM2 and Pab1-K131Q-RRM1-RRM2 (pKB351) bound to Cy5-Poly(A)20-Cy5 mRNA. RNA binding was quantified using ImageQuant Software 8.2 from single channel fluorescent images of Cy5-Poly(A)20 EMSA gels for each Pab1 protein fragment. Bands were normalized to the relative known RNA band (4000 µg) and the percentage of RNA bound was calculated. Analysis of three independent biological replicates. Error bars indicate SEM. *$p<0.05$ determined using a two-way ANOVA test.

A

Hydrogen bond from PDB residue 131

Wild type

Donor	Acceptor	Distance (Å)	Type
D0131-LYS NZ	D0208-ASP OD2	3.68	SS

Mutant

Donor	Acceptor	Distance (Å)	Type
D0131-ARG NH1	D0206-GLU OE1	3.80	SS
D0131-ARG NH1	D0208-ASP OD2	3.11	SS
D0131-ARG NH1	D0251-GLU OE2	3.39	SS
D0131-ARG NH2	D0206-GLU OE1	2.60	SS
D0131-ARG NH2	D0208-ASP OD2	3.78	SS

M: main chain, S: side chain

B

Hydrogen bond from PDB residue 131

Wild type

Donor	Acceptor	Distance (Å)	Type
D0131-LYS NZ	D0208-ASP OD2	3.68	SS

Mutant

Donor	Acceptor	Distance (Å)	Type
D0131-GLN N	D0131-GLN OE1	2.57	MS

M: main chain, S: side chain

Figure S3: mutations affect predicted hydrogen bond formation. A) Missense 3D predictions of Hydrogen bond formation for Pab1 WT and Pab1-K131R. Amino groups present in the arginine side chain increase the number of predicted interactions between D206 and D208 side chains. B) Hydrogen bond prediction for Pab1-K131Q show no putative interactions between other residues. A prediction of self-interaction between the residue main (M) carboxylic acid and side chain (S) is shown.
